# Prevalence of Anterior Inferior Iliac Spine Dysmorphism and Development of a Novel Classification System: An Anatomic Study of 1,797 Cadaveric Specimens

**DOI:** 10.3389/fsurg.2020.587921

**Published:** 2021-01-15

**Authors:** Derrick M. Knapik, Chad M. Fortun, Christopher R. J. Schilf, Shane J. Nho, Michael J. Salata

**Affiliations:** ^1^Department of Orthopaedic Surgery, University Hospitals Cleveland Medical Center, Cleveland, OH, United States; ^2^Case Western Reserve University School of Medicine, Cleveland, OH, United States; ^3^Carolina Sports Medicine and Orthopaedic Specialists, Wilmington, NC, United States; ^4^Midwest Orthopaedics at Rush University, Chicago, IL, United States

**Keywords:** subspine impingement, hip, cadaver, anatomy, hip dysplasia

## Abstract

**Purpose:** Subspine impingement occurs due to a morphologically abnormal anterior inferior iliac spine (AIIS), capable of causing impingement against the distal femoral neck. The purpose of this investigation was to determine the prevalence of AIIS dysmorphism based on specimen sex, race, and age, while introducing a novel anatomic-based classification system.

**Methods:** A total of 1,797 adult cadaveric specimens (*n* = 3,594 hemipelvises) were analyzed. AIIS with the potential for subspine impingement (SSI) was recorded in each specimen by two independent authors. Specimens with AIIS dysmorphism were then reexamined to determine SSI subtype using a novel descriptive anatomic classification system.

**Results:** AIIS dysmorphism was present in 6.4% (*n* = 115 of 1,797 specimens) of specimens and 5.2% (*n* = 186 of 3,594) of hemipelvises. Dysmorphism was significantly more common in male specimens (*p* = 0.04) and African–American specimens (*p* = 0.04). No significant overall difference in prevalence was appreciated based on specimen age (*p* = 0.89). Subtype classification found that 67% of hemipelvises possessed a columnar type AIIS, 30% were bulbous and 3% hook type. Males possessed a significantly higher prevalence of columnar type AIIS dysmorphism (*p* < 0.001). No significant overall differences in anatomic classification were appreciated based on race (*p* = 0.12) or when analyzed based on age (*p* = 0.34).

**Conclusion:** AIIS dysmorphism was present in 6.4% of the 1,797 cadaveric specimens evaluated. African-American and male specimens possessed significantly higher prevalence of AIIS dysmorphism, with no significant difference based on specimen age. Columnar type AIIS dysmorphism was most common. Anatomic classification was not significantly different based on specimen race or age.

**Level of Evidence**: Case Series, Level IV.

## Introduction

Morphologic changes to the femoral head or acetabulum characteristic of femoroacetabular impingement (FAI) result in abnormal contact forces within the hip joint and represent a well-recognized cause of hip pain and intra-articular pathology (1, 2). In contrast, extra-articular disorders of the hip secondary to subspine, iliopsoas, ischiofemoral, and greater trochanteric impingement are largely under-reported and under-recognized sources of hip pain (3–6). However, extra-articular impingement represents the second most common cause for failed hip preservation surgery requiring revision behind insufficient FAI resection (7). Subspine impingement (SSI) is characterized by a morphologically abnormal and prominent anterior inferior iliac spine (AIIS) capable of causing abnormal extra-articular contact and stress from abutment of the AIIS against the distal femoral neck during hip flexion and internal rotation (3, 8–13). Isolated SSI has been shown to be a potential source of severe hip pain and disability, necessitating operative decompression in the setting of failed non-operative management (6).

The anterior inferior iliac spine arises above the level of the anterior–superior acetabular rim (9). Morphologic abnormalities to the AIIS capable of causing SSI have been shown to occur primarily in younger males (14–23 years of age) participating in sporting activities secondary to trauma to AIIS (4, 8, 9, 12, 14–18). AIIS dysmorphism has also been reported to occur the setting of over-correction following periacetabular osteotomy or as a developmental variant seen in association in patients with acetabular retroversion (8–11, 19, 20). Patients with SSI classically present with anterior hip and groin pain aggravated by straight hip flexion, with decreased terminal hip flexion and internal rotation and focal tenderness over the AIIS on physical examination (8, 9, 11, 14).

Studies examining the etiology and management of SSI are limited primarily to case reports and small case series, while little is known regarding the prevalence of AIIS dysmorphism based on patient sex, race, and age. The purpose of this investigation was to define the prevalence of AIIS dysmorphology capable of causing SSI in a large population of osseous cadaveric specimens, while introducing a novel anatomic-based classification system for AIIS anatomy. The authors hypothesized a small overall prevalence of AIIS dysmorphism, occurring primarily in male patients with no differences in prevalence or anatomic subtypes based on race or age at the time of death.

## Materials and Methods

Cadaveric hemipelvises from the Hamann-Todd Human Osteological Collection at the Cleveland Museum of Natural History were analyzed. The collection contains over 3,000 complete, disarticulated human skeletons gathered between 1912 and 1938, cataloged for age, sex, and race in a large, well organized database. As this is a publically available collection, Institutional Review Board approval was not required. Inclusion criteria included specimens with intact bilateral hemipelvises and preserved subspine anatomy in human skeletons over the age of 18 years at the time of death. Specimens were excluded if they were missing either hemipelvis, under 18 years of age at the time of death, possessed evidence of trauma to the hemipelvis or AIIS, or had bone loss to the hemipelvis or AIIS secondary to excessive wear or damage. All available specimens meeting inclusion criteria were included within the analysis.

**Figure 1:**
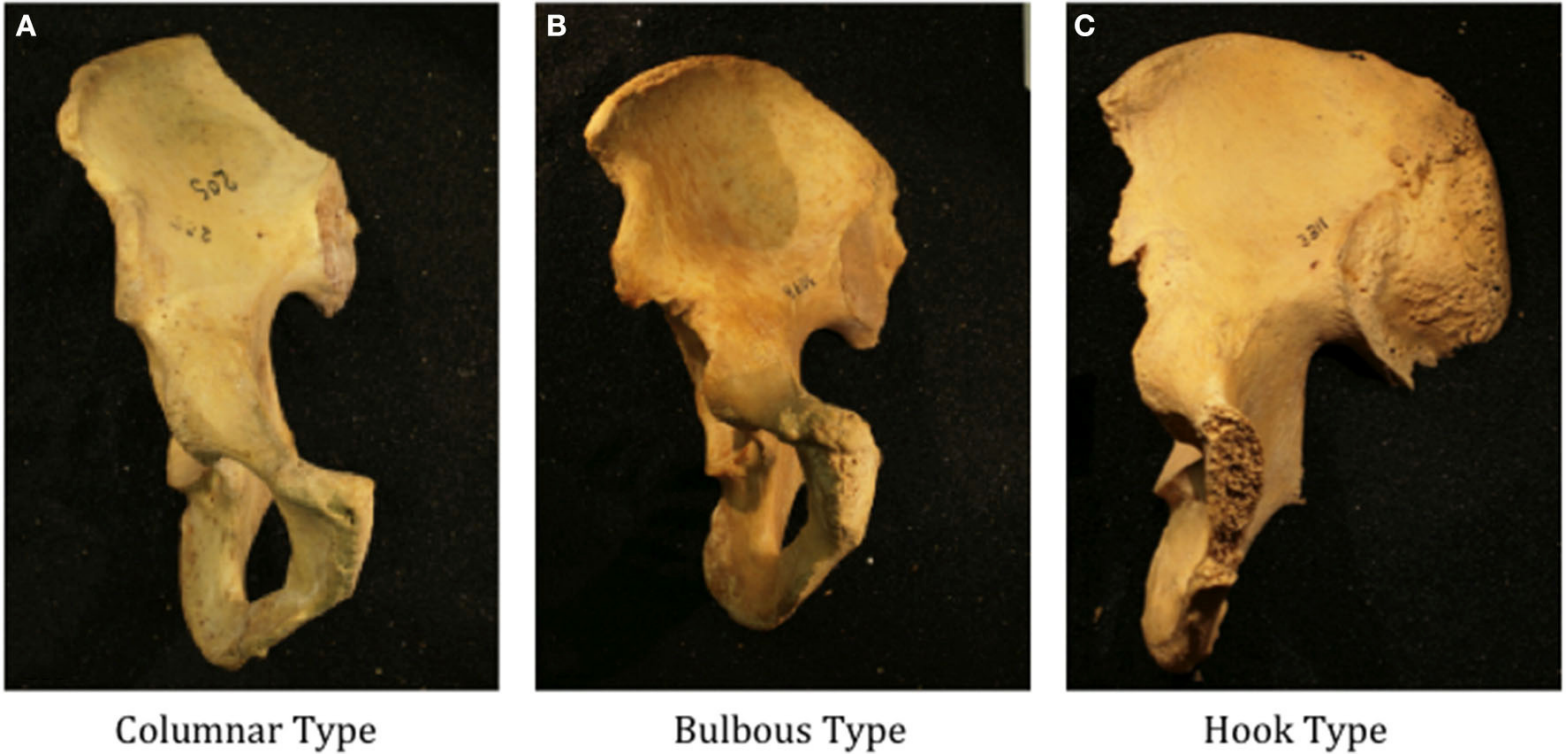
Representative cadaveric hemi pelvises demonstrating subspine impingement subtypes based on anatomic morphology: **(A)** columnar, **(B)** bulbous and **(C)** hook.

The right and left hemipelvis was evaluated by two independent authors [C.M.F., C.R.J.S.] to determine the presence of AIIS dysmorphism with the potential for SSI, defined by anterolateral extension of the AIIS beyond the sagittal plane of the acetabulum or inferior extension below the level of the acetabular sourcil (9, 11). If any discrepancies were present between the authors on the presence AIIS dysmorphism, the senior author [M.J.S.], a fellowship trained orthopedic surgeon with a specialization in the treatment of hip impingement, was asked to examine the hemipelvis to settle any disagreement. Cadaveric specimens with AIIS dysmorphism were then reexamined by the two independent authors [C.M.F., C.R.J.S.] to determine SSI type using a novel descriptive anatomic classification system based on three anatomic subtypes: columnar, bulbous or hook type morphologies (Figures 1A–C). Columnar type AIIS morphology consisted of inferior continuation of the AIIS into the anterosuperior acetabular rim without evidence of a lip or shelf. Bulbous type was defined by anterolateral extension of the AIIS with a clear endpoint to the anterior column where the anterosuperior acetabular shelf was maintained without significant inferior projection below the level of the sourcil. Hook type morphology was defined by anterolateral extension of the AIIS to the sagittal plane of the acetabulum and inferior projection to the level of the sourcil. Any discrepancies between the two authors regarding anatomic classification were again settled by consultation with the senior author to determine the final recorded classification.

Intra-class correlation coefficient values were calculated to determine Inter-observer reliability for the presence of AIIS dysmorphism in all included specimens and anatomic subtype classification in all specimens with AIIS dysmorphism capable of causing SSI. Intra-observer reliability testing was calculated for the presence of AIIS dysmorphism and anatomic classification by a single author [C.M.F.] on a total of 20 specimens (*n* = 40 hemipelvises) two weeks following initial evaluation. The authors considered an ICC of <0.4 to be poor, 0.4–0.75 to be fair to good, and >0.75 to be excellent, based on established recommendations (21, 22). Specimens were grouped based on age of death into seven separate groups: 18–29, 30–39, 40–49, 50–59, 60–69, 70–79, and 80+. Differences in the prevalence of AIIS dysmorphism based on specimen race, sex, and laterality were analyzed using a Chi-square analysis. One way analysis of variance (ANOVA) was utilized to evaluate AIIS dysmorphism prevalence based on age, while also analyzing anatomic classification based on sex, race, and age with *post-hoc* comparisons using the Tukey test. Values were considered statistically significant where *p* < 0.05. All analyses were performed using SPSS statistical software (V. 25.0, IBM Corporation, Armonk, NY).

## Results

A total of 2,612 cadaveric specimens were initially evaluated. Thirty-one percent (*n* = 815) of specimens were excluded due to missing skeletal remains preventing evaluation of the right and left hemipelvis (*n* = 712 specimens), specimens under the age of 18 at the time of death (*n* = 83 specimens) or evidence of bone loss or damage to the hemipelvis or AIIS preventing reliable evaluation (*n* = 20 specimens). A total of 1,797 complete human pelvises (*n* = 3,954 hemipelvises) meeting inclusion/exclusion criteria were analyzed. The mean age at the time of death for included specimens was 47.3 ± 15.6 years (range, 18–105 years), with Caucasian specimens (52.1 ± 14.9 years) found to be significantly older at the time of death compared to African–American specimens (42.1 ± 14.8 years; *p* < 0.001; Table 1) AIIS dysmorphism was present in 6.4% (*n* = 115 of 1,797 specimens) of specimens and 5.2% (*n* = 186 of 3,594) of hemipelvises. Unilateral dysmorphism was appreciated in 2.4% (*n* = 44 of 1,797) of specimens with bilateral dysmorphism in 4% (*n* = 71 of 1,797 specimens) of the total cohort and 62% (*n* = 71 of 115) of specimens with evidence of dysmorphism. Determination of AIIS dysmorphism between the two independent authors yielded an ICC value of 0.76, while intra-observer reliability testing in a single author resulted in an ICC value of 0.76, both indicating excellent reliability. Disagreement on the presence or absence of abnormal AIIS morphology was present in 1.4% (*n* = 26) of specimens requiring consultation with the senior author. No significant difference in AIIS dysmorphism was appreciated based on laterality (*p* = 0.19; Table 2) The prevalence of AIIS morphology was significantly higher in male specimens when compared to female specimens (*p* = 0.04). AIIS dysmorphism was significantly more common in African–American specimens when compared to Caucasians (*p* = 0.04). When analyzed based on age, no significant difference was appreciated when comparing all specimens based on age groups (*p* = 0.89) or when all age groups were separately analyzed in male (*p* = 0.73), female (*p* = 0.64), African–American (*p* = 0.91), and Caucasian (*p* = 0.97) specimens.

**Table 1:** Demographics of specimen evaluated during investigation.

		**Number of specimens (% of Total)**	**Mean age at time of death (± SD)**	***p*-value**
Sex	Male	1,500 (83%)	49.2 ± 15.1	<0.001
	Females	297 (17%)	44.9 ± 17.6	
Race	Caucasian	1,145 (64%)	52.1 ± 14.9	
	African–Americans	647 (36%)	42.1 ± 14.8	<0.001^*^
	Asian	5 (0.3%)	38.4 ± 14.4	

* *Post-hoc testing demonstrating significant difference in age between Caucasians and African–Americans (p < 0.001), with no statistically differences between Asian–Americans and Caucasians (p = 0.98) or African–Americans (p = 0.84)*.

**Table 2:** Analysis of anterior inferior iliac spine dysmorphism.

		**Number of specimens**	**Number of specimens with AIIS dysmorphism**	**% of AIIS dysmorphism**	***p*-value**
Race	African–American	647	52	8.0	0.04^*^
	Caucasian	1,144	62	5.4	
	Asian	5	1	20	
Sex	Male	1,500	104	6.9	0.04^*^
	Female	297	11	3.7	
Laterality	Right	1,797	97	5.4	0.19
	Left	1,797	89	5.0	
Age	18–29	206	14	6.8	
	30–39	326	23	7.0	
	40–49	421	27	6.4	
	50–59	371	27	7.2	0.89
	60–69	277	15	5.4	
	70–79	145	7	4.8	
	80+	51	2	3.9	

* *Chi-square analysis performed between African–American and Caucasian specimens due to limited number of Asian specimens*.

Anatomic evaluation of specimens based on AIIS morphologic classification found that 67% (*n* = 124 of 186) of hemipelvises possessed a columnar type AIIS, 30% (*n* = 56 of 186) were bulbous and 3% (*n* = 6 of 186) hook type. Inter-class reliability testing resulted in an ICC value of 0.85, while intra-class reliability testing yielded a value of 0.77, both demonstrating excellent reliability. Analysis between males and females showed a statistically significant difference overall between males and females (*p* = 0.03), with *post-hoc* testing demonstrated males to possess a significantly higher prevalence of columnar type AIIS dysmorphism compared to females (*p* < 0.001; Table 3) Males were found to possess a significantly higher prevalence of columnar type AIIS dysmorphism compared to bulbous type (*p* = 0.03), while no analysis comparing columnar and bulbous types to hook type specimens were able to be reliably performed due to low numbers of hook type specimens in males and overall specimens in females. No significant overall differences in anatomic classification were appreciated based on race (*p* = 0.12). Furthermore, no significant differences in classification were appreciated overall based on age (*p* = 0.34) or when age groups were separately analyzed based on sex (males, *p* = 0.24; females, *p* = 0.13) or race (African–American, *p* = 0.55; Caucasian, *p* = 0.21).

**Table 3:** Anterior inferior iliac spine anatomic classification.

		**Columnar**	**Bulbar**	**Hook**	***p*-value**
Sex	Male	107	55	6	0.03
	Female	17	1	0	
Race	Caucasian	70	27	2	0.12^*^
	African–American	53	29	3	
	Asian	1	0	1	
Age	18–29	12	13	0	
	30–39	22	15	0	
	40–49	32	12	3	
	50–59	31	9	1	0.34
	60–69	14	5	2	
	70–79	10	1	0	
	80+	3	1	0	

* *One-way analysis of variance performed between African–American and Caucasian specimens due to limited number of Asian specimens*.

## Discussion

The purpose of this investigation was to determine the prevalence of AIIS dysmorphism using a cadaveric collection based on specimen sex, race, and age, while reporting a novel anatomic-based classification system. This investigation found that the prevalence of AIIS dysmorphism was 6.4% in the 1,797 specimens evaluated, with significantly higher prevalence of AIIS dysmorphism with the potential for SSI in African-American and male specimens. No significant difference in the prevalence of AIIS dysmorphism was appreciated based on patient age. Using our novel classification system, columnar type AIIS dysmorphism was most common anatomic subtype appreciated. Males were found to possess significantly higher rates of columnar dysmorphism compared to bulbous type, while anatomic classification was not found to be significantly different based on specimen race or age.

The prevalence of AIIS dysmorphism was significantly higher in male specimens when compared to female specimens. This finding is in contrast to the findings from previous investigations examining AIIS dysmorphology prevalence based on sex. The study by Hetsroni et al. (10) characterized and defined AIIS morphology using three-dimensional computed tomography reconstructions of 53 hips and reported no sex-based predisposition to AIIS dysmorphology (*n* = 28 males, *n* = 25 females). In addition, Wong et al. (3) reported that sex was not a risk factor for the development of AIIS dysmorphology capable of causing impingement in their evaluation of 54 patients with symptomatic hip impingement. Meanwhile, the case series by Nwachukwu et al. (6) examining outcomes following arthroscopic decompression of isolated SSI reported that all 33 patients in their cohort were women, leading the authors to conclude that despite the balanced sex-based distribution previously reported by Hetsroni et al. (10), women may be more likely to have symptomatic isolated AIIS-related extra-articular hip impingement. The predilection for extra-articular hip impingement in females was further corroborated by Torriani et al. (23) who reported ishiofemoral impingement to occur exclusively in women, while Ricciardi et al. (24) found that 85% of patients presenting with all forms of extra-articular hip impingement (subspine, iliopsoas, ischiofemoral, and greater trochanteric) were females. While it has been proposed that different morphologic characteristics inherent within the female hip, namely increased femoral and acetabular anteversion (25), may predispose females to the development of symptomatic SSI, the prevalence of AIIS dysmorphism leading to SSI based on sex remains largely unknown and merits further investigation.

Increasing age with secondary osteoarthritic changes was not shown to be associated with a higher prevalence of AIIS dysmorphism or anatomic subtype capable of causing SSI. These findings are in agreement with the results reported by Wong et al. (3) in which age was not found to be a significant risk factor for the development of extra-articular hip impingement with increasing age. In our cohort, a higher prevalence of AIIS dysmorphism was found in African-American specimens, who possessed a significantly lower mean age at the time of death when compared to Caucasian specimens. Moreover, the retrospective investigation by Yoo et al. (26) of 427 patients with mechanical hip pain vs. 259 control patients found that that youth was the only significant risk factor for possessing AIIS dysmorphology capable of causing SSI. Etiological factors leading to AIIS dysmorphism with the potential to cause SSI include post-traumatic causes following physeal avulsion or chronic traction hypertrophy, post-surgical following overcorrection following periacetabular osteotomy, or developmental abnormalities seen in association with patient with acetabular retroversion (14, 18, 20). As such, development of AIIS dysmorphology does not appear to correlate with increasing age and associated osteoarthritic changes (9, 27–29). In addition, our investigation is the first to examine AIIS dysmphology prevalence based on race, necessitating further research to determine the impact of racial differences on AIIS morphology differences potentially leading to SSI.

This investigation utilized a novel anatomic based classification system to characterize AIIS dysmorphology. The most commonly utilized classification system describing AIIS dysmophology was reported by Hetsroni et al. (10) in their evaluation of 53 hips from 53 patients with hip impingement using 3-dimensional CT reconstructions. The proposed a classification system by Hetsroni et al. (10) was based on three types of morphological AIIS variants: Type 1, consisting of a smooth ilium wall between the AIIS and the acetabular rim; Type 2, with the AIIS extending to the level of the acetabular rim; and Type 3, AIIS extending distally to the acetabular rim. The authors found that Type 1 variants did not contribute to hip impingement, while Type 2 and 3 variants were associated with decreased hip flexion and internal rotation secondary to contribution of the AIIS to impingement with the femoral neck at terminal hip motions. However, other authors have proposed that Type 1 hips may cause impingement against the distal femoral neck during excessive hip flexion, as is seen in ballet and high kick dancing (11). Meanwhile, the investigation by Karns et al. (30) retrospectively analyzed AIIS morphology in 145 patients on anteroposterior and false profile radiographs, reporting no correlation between hip flexion, and internal rotation based on the AIIS classification type proposed by Hetsroni et al. (10) Moreover, Balazs et al. (31) found no difference in the distribution of Type 1 vs. Types 2 and 3 AIIS morphology when comparing symptomatic and asymptomatic patients, further questioning the association of AIIS in morphology in clinical extra-articular hip impingement. In addition, the authors reported difficulty distinguishing between Type 1 and Type 2 morphologies due to the confluence of the ilium wall and acetabular rim resulting in a lack of a distinct separation (31). As such, we have proposed a new anatomic classification system based on anatomic appearance as a means of providing a simpler method of classifying AIIS dysmorphology, however the correlation between this novel anatomic subtype classification system on predicting symptomatic SSI warrants further investigation in a modern patient population.

### Limitations

This study was not without limitations. No clinical information was available to determine if any specimen possessed hip pain secondary to SSI while alive. As such, despite the presence of AIIS dysmorphia, the authors are unable to determine if patients were symptomatic or asymptomatic based on the presence of AIIS dysmorphism or anatomic subtype. Due to the use of dry cadaveric specimens, the authors are unable to assess the contribution of the surrounding soft tissue structures about the hip which have been shown to be compressed through interposition or placed under tension during range of motion, contributing to hip pain and diagnosis of SSI (32). Prior studies have reported a high prevalence of labral pathology in patients presenting with SSI secondary to AIIS impingement on the hip capsule and acetabular-labral complex, however the author are unable to account for the contribution of these soft tissue components (6). While femoral version and hip motion have recently been suggested to influence hip internal rotation, these parameters were not evaluated in this investigation (13). In addition, femoral anteversion and valgus have been shown to create distinct motion patterns (decreased adduction and external rotation), causing impingement to occur between a normally shaped AIIS and the greater trochanter or the anteroinferior femoral neck, however these potentially contributing variables were not assessed (33). While three racial types were evaluated, the authors were unable to perform any meaningful statistical analysis using Asian–American due to the small sample of specimens. Moreover, the prevalence of AIIS dysmorphism in other racial population is unknown and cannot be generalized based on the findings from this investigation. Abnormal AIIS morphology has also been identified as a cause of the radiographic “cross-over” sign, creating the potential for over diagnosing cranioacetabular retroversion on pelvis radiographs (34). However, this phenomenon was not accounted for as no radiographs were obtained during the course of the investigation.

In conclusion, AIIS dysmorphism was present in 6.4% of the 1,797 cadaveric specimens and 5.2% of 3,594 hemipelvises evaluated. Clinically, the findings from this investigation provide clinicians with a novel and simpler method of characterizing AIIS dysmorphology, with the prevalence of dysmorphism being significantly higher in African–American and male specimens, with no significant difference based on specimen age. Columnar type AIIS dysmorphism was most common, with males possessing significantly higher rates of columnar dysmorphism compared to bulbous type, while anatomic classification was not significantly different based on specimen race or age. Future investigations examining of impact of this novel anatomic subtype classification system on the development and treatment of symptomatic impingement as well as the prevalence of AIIS dysmorphism in a modern population are warranted.

## Data Availability Statement

The raw data supporting the conclusions of this article will be made available by the authors, without undue reservation.

## Author Contributions

DK: concept design, data analysis, manuscript writing, and manuscript finalization. CF: concept design, data collection, and manuscript editing. CS: data collection and manuscript editing. SN: concept design and manuscript editing. MS: concept design, data analysis, and manuscript editing. All authors contributed to the article and approved the submitted version.

## Conflict of Interest

The authors declare that the research was conducted in the absence of any commercial or financial relationships that could be construed as a potential conflict of interest.
